# A preliminary study of sleep spindles across non-rapid eye movement sleep stages in children with autism spectrum disorder

**DOI:** 10.1093/sleepadvances/zpac037

**Published:** 2022-10-20

**Authors:** Midori Kawahara, Kuriko Kagitani-Shimono, Kumi Kato-Nishimura, Noboru Ohki, Masaya Tachibana, Takafumi Kato, Masako Taniike, Ikuko Mohri

**Affiliations:** Department of Child Development, United Graduate School of Child Development, Osaka University, Suita, Osaka, Japan; Department of Pediatrics, Osaka University Graduate School of Medicine, Suita, Osaka, Japan; Department of Child Development, United Graduate School of Child Development, Osaka University, Suita, Osaka, Japan; Department of Child Development, United Graduate School of Child Development, Osaka University, Suita, Osaka, Japan; Department of Pediatrics, Osaka University Graduate School of Medicine, Suita, Osaka, Japan; NoruPro Light Systems Incorporation, Kokubunji-shi, Tokyo, Japan; Department of Child Development, United Graduate School of Child Development, Osaka University, Suita, Osaka, Japan; Department of Pediatrics, Osaka University Graduate School of Medicine, Suita, Osaka, Japan; Department of Neuroscience and Oral Physiology, Graduate School of Dentistry, Osaka University, Suita, Osaka, Japan; Department of Child Development, United Graduate School of Child Development, Osaka University, Suita, Osaka, Japan; Department of Pediatrics, Osaka University Graduate School of Medicine, Suita, Osaka, Japan; Department of Child Development, United Graduate School of Child Development, Osaka University, Suita, Osaka, Japan; Department of Pediatrics, Osaka University Graduate School of Medicine, Suita, Osaka, Japan

**Keywords:** autism spectrum disorder, sleep spindle, thalamocortical network

## Abstract

**Study Objectives:**

Sleep spindles play a crucial role in multiple neuronal network functions. Initiation and termination of spindles are regulated by the thalamic reticular nucleus and thalamocortical network, and the spindle can be an index for brain organization. We conducted a preliminary study of the parameters of sleep spindles, focusing on sleep-stage temporal distribution in children with autism spectrum disorder (ASD) with normal intelligence/developmental quotients.

**Methods:**

We performed overnight polysomnography in 14 children with ASD (4–10 years) with normal full-scale intelligence quotient/developmental quotient (≥75) and 14 community samples (CS) of children. Sleep stages were scored according to the Rechtschaffen and Kales criteria. Spindle parameters were quantified and compared between these groups and the identified subgroups.

**Results:**

Sleep parameters did not differ between the ASD and CS groups, except for a higher rate of rapid eye movement (REM) sleep duration in ASD. Spindle parameters did not significantly differ between the groups, but spindle density was distributed in a broader range in the ASD group. Five children with ASD had a higher spindle density in stage 3 than in stage 2. The ratio of spindle density in stage 3 to that in stage 2 (stage 3/2 ratio) was significantly higher in ASD than in CS cases.

**Conclusions:**

The lower spindle density in stage 2 and relatively higher density in stage 3 in children with ASD may represent an abnormal generation of spindles due to insufficient maturation of the thalamic reticular nucleus and thalamocortical network.

**Statement of Significance**

This preliminary study examined the parameters of sleep spindles across non-rapid eye movement (non-REM) sleep stages in children with autism spectrum disorder (ASD). As characteristics of the spindle may vary with age and cognitive function, we selected children with the age range of childhood (4–10 years) and normal intelligence quotients. We found that the appearance of sleep spindles widely varied in ASD children compared with the community samples, suggesting the heterogeneous nature of ASD. In addition, the abnormal distribution of sleep spindles in sleep stages 3 and 4 may reflect an insufficient maturation of the thalamic reticular nucleus and the thalamocortical network, in especially younger children and those with lower intelligence quotients. The analysis of sleep spindles may provide an additional tool for understanding the pathophysiology of ASD.

## Introduction

Sleep spindles are waxing and waning waves with a frequency of 10–15 Hz may last for 0.5–2 s [1, 2]. They are characteristic transient features of the sleep electroencephalogram (EEG). Based on the American Academy of Sleep Medicine (AASM) manual defining the scoring rules of sleep and associated events in children, two types of sleep spindles, namely fast (12.5–14.75 Hz), and slow (10.0–12.75 Hz) spindles, have distinct temporal and topographical profiles [3]. Sleep spindles are considered hallmarks of stage 2 non-rapid eye movement (non-REM) sleep [4] determined using the Rechtschaffen and Kales (R&K) criteria [5], and the spindle density is significantly higher during sleep stage 2 than during stage 3 [6]. The spindle density increases with age during childhood, peaking around adolescence; then, it declines across adulthood [7]. Sleep spindles are generated by the interplay of the thalamic reticular nucleus (TRN), which is one of the most prominent brain nuclei that synthesizes gamma-aminobutyric acid (GABA) [8], with other thalamic nuclei. Then, the sleep spindles are sustained and relayed to the cortex by thalamothalamic and thalamocortical (TC) loops [9].

The roles and significance of sleep spindles are a topic of interest in the field of neuroscience. Sleep spindles reportedly maintain sleep continuity [10] and regulate arousal [11]. In addition, sleep spindles may play a crucial role in multiple neuronal network functions, such as memory consolidation and plasticity via dynamic alterations in synaptic plasticity [12], and can be considered as a proxy measure for the individual’s learning potential [13]. For example, human studies have reported an increase in spindle density and/or activity after learning [6]. Enhanced spindle activity was observed after the acquisition of both declarative memory tasks and procedural motor skills [14, 15]. This may be related to the evidence that spindle activation is highly correlated with the intelligence quotient (IQ) [13].

Studies have investigated whether the emerging pattern of sleep spindles could be an indicator of the development of psychiatric and neurodevelopmental disorders. Sleep spindle deficits have been confirmed in several groups of patients with chronic and medicated schizophrenia [16]. Children aged <18 months with intellectual disabilities have fewer sleep spindles [2]. In children with attention-deficit/hyperactivity disorder (ADHD), the power in the frequency of slow sleep spindles is higher in frontal regions, and this is even more the case when they also have autism spectrum disorder (ASD) [17]. Furthermore, more severe ADHD symptoms are correlated with the weaker activity of fast sleep spindles [18].

ASD is a complex developmental condition involving persistent challenges with social communication, restricted interests, and repetitive behavior (Diagnostic and Statistical Manual of Mental Disorders 5th Edition; DSM-5). ASDs are complex, pervasive, heterogeneous, and multifactorial neurodevelopmental conditions [19]. Neurochemical investigations of ASD pathogenesis suggest that serotoninergic, dopaminergic, noradrenergic, and GABAergic systems are fundamentally affected in various ways [20]. Furthermore, many researchers have reached a broad consensus that the main pathology of ASD resides in abnormal connectivity between brain regions, including the cortex, thalamus, and brainstem [21]. Therefore, such neurotransmitter system and connectivity abnormalities may affect spindle appearances in individuals affected by ASD. Different findings exist concerning spindles in different sampled demographics and brain regions. For instance, Limoges et al. [22] found fewer sleep spindles over the central regions in adults with ASD. Considering children, Tessier et al. [23] reported a lower sleep spindle density in the prefrontal area of patients of ASD with normal IQ, Farmer et al. [24] found decreased spindle density and duration on the central and frontal spindles in a large cohort of ASD including patients with lower IQ, whereas Maski et al. [25] reported no difference in the sleep spindles on Cz electrode between children with ASD with normal IQ and typically developing (TD) children. Various alterations have been found; however, these studies included different ages and IQs, which could be confounding factors. Analyzing different electrode signals could also affect the result. In children with ASD, decreased spindle density is observed only in the frontal lobe.

In this study, we analyzed the parameters of sleep spindles, focusing on the temporal distribution during non-REM sleep in Japanese children with ASD with normal or high IQ. Furthermore, we examined the sleep stage temporal distribution of the spindle upon analysis of the raw polysomnography (PSG) data. Although sleep spindles are considered hallmarks of stage 2 non-REM sleep, we also analyzed sleep spindles in stages 3 and 4. Analyzing sleep spindles in different stages may provide a new point of view on sleep spindles in children with ASD and facilitate our understanding of ASD pathogenesis.

## Methods

### Participants

Children with ASD were recruited at the pediatric clinic of the Osaka University Hospital. We applied PSG to those who had complaints of snoring to rule out sleep disorder in children with ASD. We used PSG whose obstructive apnea-hypopnea index (OAHI) value was <1. A diagnosis of ASD was made by four pediatric neurologists (K.K.S., M.Tac., M.Tan., and I.M.) according to the DSM-5 criteria. Older cases whose initial diagnoses were made according to the DSM-IV-TR, prior to the release of DSM-5 in 2013, were rediagnosed using the DSM-5 criteria. Moreover, it was confirmed using the Autism Diagnostic Observation Schedule-Generic (ADOS-G) and the ADOS-2 (ADOS-2) manual [26], which evaluates core ASD symptoms and has been the gold standard for ASD diagnosis. The full-scale intelligence quotient (FSIQ) and developmental quotient (DQ) were assessed using the Wechsler Intelligence Scale for Children (WISC-III or IV) and the Kyoto Scale of Psychological Development [27], respectively. The Child Behavior Checklist [28], a comprehensive assessment of behavioral problem areas, was completed by the caregivers. In this study, children with ASD, those who had FSIQ/DQ scores <75, those who were taking psychoactive medicines regularly, and those with comorbid epilepsy were excluded. All patients with ASD had normal-range language development.

Community samples (CS) of children were recruited through public newsletters distributed in the Osaka prefecture. The samples were confirmed using both observations by pediatric neurologists and history-taking, which included developmental history, the absence of any diagnosis of developmental disorders, and non-receipt of educational support. The parent-reported Japanese versions of the ADHD Rating Scale [29] and social responsive scale [30, 31] were used for all CS of children to confirm that their scores were within the normal range. Although the WISC-III or IV was not used in this CS group, we confirmed normal performance by applying the four tests of the Cambridge Neuropsychological Test Automated Battery (Cambridge Cognition, UK), i.e. Intra-extra Dimension Set Shift (an analog of Wisconsin Card Sorting), Spatial Working Memory, Stocking of Cambridge (an analog of Tower of London), and Stop Signal Task evaluating executive functions. After PSG, children with OAHI values >1 and those who were suspected to have obstructive apnea were excluded from the study. This study was approved by the Ethics Committee of the Pedagogy course of the Graduate School of Human Sciences, Osaka University (approval no. 12168). All the study procedures were conducted in accordance with the ethical standards of the Declaration of Helsinki.

In total, 14 children with ASD (age: 6.4 ± 1.7 years, range: 4–10 years, sex: 12 boys, two girls; FSIQ: 103.3 ± 15.7, range: 75–122; DQ: 95.6 ± 10.5, range: 89–119) and 14 CS of children (age: 7.6 ± 1.5 years, range: 4–10 years, sex: seven boys, seven girls) participated in this study. Although the ASD group included younger children and more boys, there was no significant difference in either age or sex between the ASD and CS groups (Table 1). Considering brain development, major events take place during the early postnatal, prepubertal, and adolescent periods; spindle properties are modified during both early and later phases of brain development [6]. We analyzed the age range of the prepubertal period. Moreover, we excluded patients aged >11 years because a significant difference was reported in a study comparing the spindle density between a group of patients aged 4 years and a group of patients aged 11 years [32].

**Table 1.** Demographic data and sleep macrostructure in the overnight polysomnography study

	ASD	CS	*P*	ES *d*	1−β
Number of participants	14 (12)	14 (7)	–	–	–
(Number of boys^*^)			(0.05)	–	(0.41)
Age (years)^†^	6.4 ± 1.7	7.6 ± 1.5	0.05	0.75	0.48
FSIQ (*n* = 7)^†^	103.3 ± 15.7	n.a.	–	–	–
DQ (*n* = 7)^†^	95.6 ± 10.5	n.a.	–	–	–
OAHI (number/h)^†^	0.5 ± 0.4	0.4 ± 0.3	0.08	0.28	0.11
Total sleep time (min)^†^	524.9 ± 38.7	498.3 ± 54.5	0.15	0.56	0.30
Sleep latency (min)^†^	33.4 ± 27.5	32.4 ± 18.9	0.92	0.04	0.05
Sleep efficiency (%)^†^	87.9 ± 7.8	88.9 ± 5.0	0.70	0.15	0.07
Wake after sleep onset (min)^†^	33.4 ± 27.3	18.0 ± 12.3	0.06	0.73	0.46
Stage 1 duration (%)^†^	13.3 ± 5.6	10.6 ± 7.7	0.30	0.40	0.18
Stage 2 duration (%)^†^	38.0 ± 8.5	40.9 ± 9.0	0.40	0.33	0.13
Stage 3 duration (%)^†^	7.6 ± 4.8	6.4 ± 2.3	0.40	0.32	0.13
Stage 4 duration (%)^†^	17.9 ± 6.4	22.3 ± 4.6	0.05	0.79	0.52
REM sleep duration (%)^†^	23.1 ± 4.4	19.8 ± 3.5	<0.05	0.83	0.56

Data are presented as means ± SD. SD, standard deviation; ASD, autism spectrum disorder; CS, community samples; FSIQ, full-scale intelligence quotient; DQ, developmental quotient; OAHI, obstructive apnea-hypopnea index; REM, rapid eye movement; *P*, *p*-value; ES *d*, effect size *d*; 1−β, power; n.a., not applicable.

^*^Fisher’s exact test.

^†^Student’s *t*-test.

### Sleep recordings

Overnight PSG was performed in the sleep laboratory of the pediatric ward of the Osaka University Hospital and at the research sleep laboratory of the Department of Dentistry of the Osaka University in children with ASD and CS of children, respectively, using the Rembrandt system (Embla, CO, USA). The PSG montage included four EEG channels (C3, C4, O1, and O2), submental electromyograms, left and right electrooculograms, electrocardiograms, chest respiratory movements, and electromyograms from the left and right tibialis muscles. In addition, cardiorespiratory monitoring, including the measurement of oronasal airflow with a thermistor and pressure sensor, pulse oximetry, rib cage and abdominal wall motion sensing using inductance plethysmography, and body position detection, was performed. All recordings started at the patients’ usual bedtime and continued until spontaneous awakening. During PSG, two children with ASD received hypnotic drugs (triclofos sodium and chloral hydrate, respectively) to fall asleep. There were no significant differences in demographic data and spindle characteristics between these two and the other children. History-taking and PSG confirmed that none of the participants were affected by sleep disorders, such as sleep apnea, restless legs syndrome, periodic limb movement disorder, severe insomnia, or narcolepsy. Sleep stages and arousal were manually scored in 30-s epochs according to the R&K criteria by five technicians and checked by a single certified benchmark scorer. Novelli et al. [33] and Scholle et al. [34] pointed out that various physiological and biological factors, such as secretion of growth hormones, were associated with non-REM sleep stages 3 and 4 in children and adolescents. We previously reported a decrease in body movement of children with ADHD in stage 3 compared to that in stage 4 [35]. Therefore, we also paid attention to the physiological differences between stage 3 and stage 4. For this reason, we adopted the R&K criteria to distinguish between non-REM sleep stages 3 and 4 in this study.

### Spindle analysis

**Figure 1.**
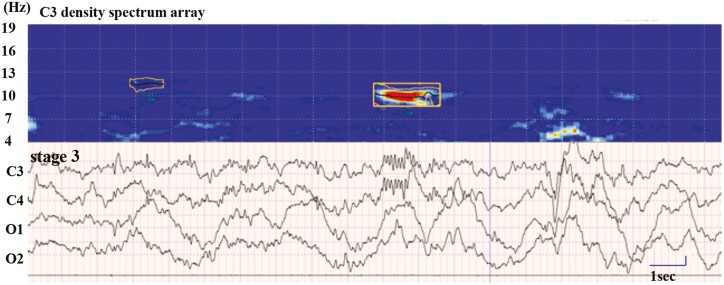
Sleep spindles recorded in stage 3 in children with ASD. Sleep spindles and the corresponding density spectrum array of C3 recorded in stage 3 in children with ASD. Sleep spindles are outlined.

**Figure 2.**
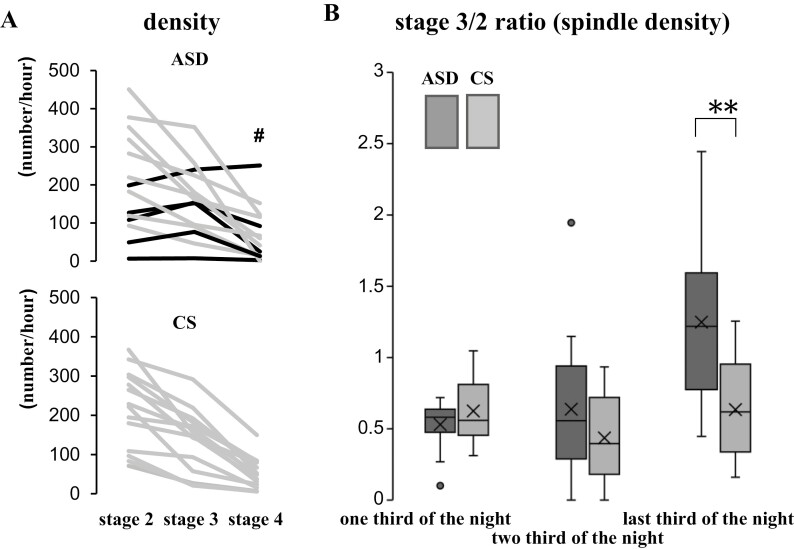
The profile of spindle density in children with ASD and CS. (A) The profile of spindle density between stages 2, 3, and 4 in each case. One child with ASD showed the highest spindle density in stage 4 (#). Black lines indicate the participants, in whom the spindle density was higher in stage 3 than in stage 2. (B) The ratio of sleep spindle density in stage 3 to stage 2 (stage 3/2 ratio) in thirds of the night in children with ASD and CS. Data were compared using the Mann–Whitney U test. Horizontal line inside the box, median; X inside the box, average; dot outside the box, outliers evaluated by the interquartile range; ASD, autism spectrum disorder; CS, community samples; stage 3/2 ratio, the ratio of spindle density in stage 3 to stage 2; **, *p*-value < .01.

Spindle density (number of spindles per h), spindle duration (ms), and spindle mean amplitude (µV) were calculated by analysis of spindles in C3 using EEG component analysis software (NoruPro Light Systems, Tokyo, Japan), where the density spectrum array of the wave with the target frequency of the band was calculated using the complex demodulation method [36] (Figure 1). The complex demodulation method readjusts the threshold parameters for detection every 60 s to extract instantaneous power in the spindle band. Spindle characteristics, such as amplitude, duration, and oscillatory frequency, are derived for each individual spindle; thus, this approach is suitable for determining spindle variability across the night and across individuals. After sleep spindles had been automatically detected, sleep spindles were identified visually and corrected manually by two researchers (K.M. and M.I.). We set the target band frequency to the range of 10–14.75 Hz with a duration of more than 0.5 s, according to the AASM manual, as 80% of children aged <13 years present two location-dependent frequency ranges for sleep spindles: 10.0–12.75 Hz over the frontal and 12.5–14.75 Hz over the central or centroparietal region [37]. After sleep spindles had been automatically detected, a manual correction was performed in a blinded manner. All parameters were calculated per total sleep time, as well as per distinct sleep stages. Based on the researcher’s observations, we conducted post-hoc analyses to estimate the temporal distribution of the spindle. To estimate the tendency of sleep spindle appearance, we calculated the spindle density, duration, and amplitude using the ratios of the values in stage 3 to those in stage 2 (stage 3/2 ratio). Moreover, we divided the data (sleep stage% and the spindle densities) by thirds of the night (Table 2, Supplementary Table 1, and Figure 2, B).

**Table 2.** Differences of spindle density in stages 2, 3, and 4 among the ASD and CS groups divided in thirds of the night

Density (number/hour)		Stage 2			Stage 3			Stage 4		
		Mean ± SD	*P*	ES *d*	Mean ± SD	*P*	ES *d*	Mean ± SD	*P*	ES *d*
				1−β			1−β			1−β
First third	ASD	214.9 ± 126.8	0.91	0.04	118.3 ± 77.9	0.43	0.29	63.7 ± 60.8	0.64	0.34
	CS	219.1 ± 107.1		0.05	142.9 ± 90.8		0.11	46.8 ± 37.8		0.13
Second third	ASD	208.2 ± 133.0	0.87	0.10	144.7 ± 118.2	0.38	0.34	67.2 ± 78.0	0.87	0.03
	CS	220.1 ± 103.2		0.06	109.2 ± 88.5		0.13	64.8 ± 63.6		0.05
Last third	ASD	199.7 ± 138.1	0.54	0.12	226.7 ± 171.2	0.14	0.66	100.1 ± 131.8	0.84	0.38
	CS	213.5 ± 98.2		0.06	136.8 ± 87.1		0.38	61.4 ± 62.2		0.15

Data are presented as means ± SD. SD, standard deviation; ASD, autism spectrum disorder; CS, community samples; *P*, *p*-value; ES *d*, effect size *d*; 1−β, power.

### Statistics

We used Fisher’s exact test to compare sex, and also the usage of hypnotic drugs during EEG. Demographic data and calculated parameters were compared between the ASD and CS groups, as well as among the ASD subgroups, using Student’s t-test, or the Mann–Whitney U test when the data were not naturally distributed and the variance between the groups was not equivalent. Correlations between EEG measures in stages 2, 3, or 4 and demographic data were tested using Pearson’s rank correlation coefficient. A one-way analysis of covariance (ANCOVA) was performed to examine the differences in the spindle parameters upon controlling for the effect of age and sex between the children with ASD and CS of children. We also conducted one-way ANCOVA to examine the effect of children with ASD and the CS of children in a stage 3/2 ratio of the spindle density controlling for the effect of age. The effect size d (ES d) and power (1−β) were calculated based on the post-hoc power analysis. All statistical analyses were performed using the SPSS statistical software package (IBM Corp., Armonk, NY, USA).

## Results

**Figure 3.**
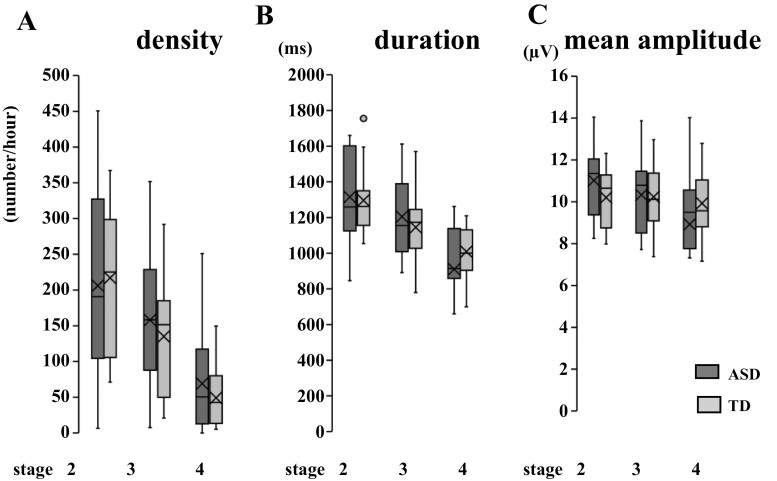
Average spindle parameters of each sleep stage in ASD and CS in C3. Average spindle density (A), duration (B), and mean amplitude (C) of each sleep stage in ASD and CS in C3. The data were compared using Student’s *t*-test. Horizontal line inside the box, median; X inside the box, average; dot outside the box, outliers evaluated by the interquartile range; ASD, autism spectrum disorder; CS, community samples.

The clinical characteristics and sleep macrostructures of the children included in this study are described in Table 1. There were no significant differences in total sleep time; sleep latency; sleep efficiency; wake time after sleep onset; percentages of stages 1, 2, 3, and 4; and OAHI between the two study groups. The percentage of REM sleep duration was significantly higher in the ASD group than in the CS group (ASD: 23.1 ± 4.4%, TD: 19.8 ± 3.5%, ES *d* = 0.83, 1−β = 0.56, *p* = 0.04). As shown in Figure 3 as well as in Tables 2 and 3, there was no significant difference between the average values of spindle parameters in C3 of the ASD and CS groups. Children with ASD and CS of children did not show a significant difference in spindle parameters after eliminating the effect of age and sex; however, the post-hoc power analysis and the effect size revealed that the differences across the groups were suggestive of a type II error.

**Table 3.** Differences in the spindle parameters in stages 2, 3, and 4 among the ASD and CS groups

		Stage 2			Stage 3			Stage 4		
		Mean ± SD	*P*	ES *d*	Mean ± SD	*P*	ES *d*	Mean ± SD	*P*	ES *d*
				1−β			1−β			1−β
Density (number/h)	ASD	206.2 ± 133.0	0.68	0.09	158.0 ± 91.7	0.24	0.27	69.1 ± 71.7	0.52	0.34
	CS	216.9 ± 98.2		0.06	135.0 ± 81.0		0.11	49.2 ± 40.6		0.15
Duration (ms)	ASD	1315.1 ± 269.4	0.62	0.08	1204.9 ± 232.1	0.85	0.27	910.2 ± 312.4	0.30	0.41
	CS	1296.5 ± 186.1		0.06	1145.3 ± 209.6		0.11	1008.9 ± 142.8		0.19
Mean amplitudes (V)	ASD	11.0 ± 1.7	0.22	0.51	10.3 ± 2.0	0.91	0.06	8.9 ± 3.2	0.29	0.40
	CS	10.2 ± 1.4		0.28	10.2 ± 1.6		0.05	9.9 ± 1.6		0.17

Data are presented as means ± SD. SD, standard deviation; ASD, autism spectrum disorder; CS, community samples; *P*, *p*-value; ES *d*, effect size *d*; 1−β, power.

As shown in Figure 3, A, the spindle density was distributed across a broader range in the ASD group (6.6–450.5/h) compared to that in the CS group (71.2–367.1/h) in all stages. However, the distribution range of duration and the mean amplitude were not substantially different between the ASD and CS groups, except for the spindle duration in stage 2. When we divided the data (sleep stage% and the spindle density) by thirds of the night, both sleep stage% and the spindle density did not differ among the children with ASD and the CS of children (Table 2, Supplementary Table 1, and Figure 2, B). Children show different characteristics from adults throughout the sleep period, such as more REM sleep in the later part of their sleep [38]. No correlations were found between the spindle parameters and FSIQ, DQ, and age in all stages, except for stage 2 density and age (Table 4). Our findings may also be associated with slower brain development.

**Table 4.** Correlation between sleep spindle parameters (stages 2, 3, and 4) and FSIQ, DQ, and age in children with ASD

		FSIQ			DQ			Age		
		*r*	*P*	1−β	*r*	*P*	1−β	*r*	*P*	1−β
Stage 2	Density^*^	0.35	0.45	0.46	−0.10	0.83	0.08	0.61	0.02^*^	0.95
	Duration^*^	0.18	0.70	0.15	−0.12	0.45	0.09	0.45	0.11	0.69
	Mean amplitude^*^	−0.08	0.87	0.07	−0.66	0.11	0.98	0.06	0.85	0.06
Stage 3	Density^*^	0.36	0.43	0.48	−0.25	0.59	0.25	0.40	0.16	0.58
	Duration^*^	0.32	0.49	0.39	−0.20	0.67	0.18	−0.05	0.86	0.06
	Mean amplitude^*^	−0.14	0.77	0.11	−0.63	0.13	0.96	0.17	0.56	0.14
Stage 4	Density^*^	0.71	0.07	0.99	−0.44	0.33	0.67	−0.08	0.78	0.07
	Duration^*^	0.43	0.34	0.65	−0.33	0.46	0.41	−0.33	0.26	0.41
	Mean amplitude^*^	0.40	0.38	0.58	−0.59	0.16	0.93	−0.15	0.61	0.12

Pearson’s rank correlation coefficient. ASD, autism spectrum disorder; FSIQ, full-scale intelligence quotient; DQ, developmental quotient; *r*, correlation coefficient; *P*, *p*-value; 1−β, power.

^*^ *P* < 0.05

As we recognized a more frequent appearance of sleep spindles in stage 3 in some ASD cases, we quantified spindle densities in each stage 2–4 to identify changes in temporal distributions of sleep spindles (Figure 2, A). Five of 14 children in the ASD group showed a higher spindle density in stage 3 than in stage 2 (Figure 2, A, indicated with black lines in the upper panel), and one child with ASD showed the highest spindle density in stage 4 (Figure 2, A, indicated with # in the upper panel), whereas all CS of children showed lower spindle densities with deeper sleep stages (Figure 2, A, lower panel).

To clarify the tendency of sleep spindle appearance, we compared the ratios of the values in a stage 3/2 ratio. ANCOVA was performed to examine the effect of children with ASD and CS of children in the stage 3/2 ratio of the spindle density controlling for the effect of age. Age was not significantly related to the stage 3/2 ratio of the spindle density F(1,25) = 1.14, *p* = .30. Children with ASD and the CS of children showed significant differences only in the stage 3/2 ratio of the spindle density in the last third of the sleep *F*(1,25) = 7.68, *p* = .01 after eliminating the effect of age (Figure 2, B).

The stage 3/2 ratios of the spindle duration and mean amplitude were not significantly different between the two groups (data not shown).

As shown in Figure 2, B, five children with ASD showed a stage 3/2 ratio ≥1 (i.e. the spindle density was higher in stage 3 than in stage 2). To evaluate the characteristics of these children with ASD whose stage 3/2 ratio of spindle density was ≥1, we divided the ASD group into two subgroups: one group whose stage 3/2 ratio of spindle density was ≥1 (stage 3/2 ≥1 group), and the other group whose stage 3/2 ratio of spindle density was <1 (stage 3/2 <1 group).

**Figure 4.**
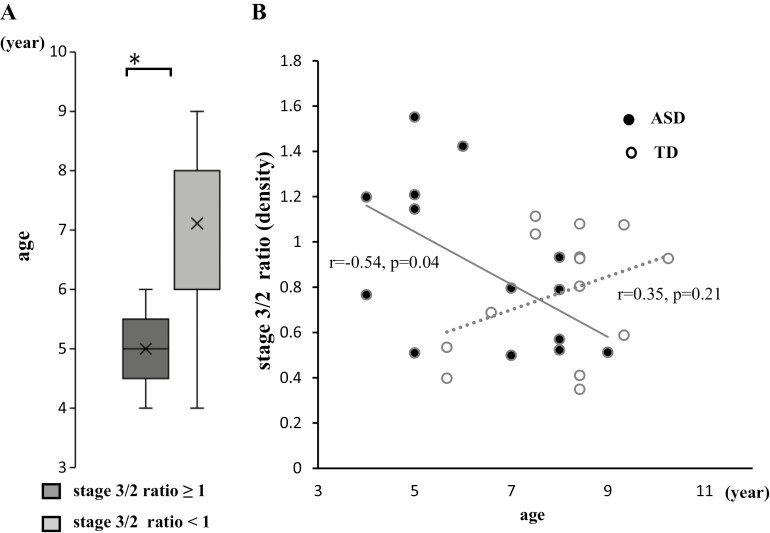
Comparison based between the stage 3/2 ratio ≥1 and stage 3/2 ratio <1 groups. (A) Comparison based on age. The data were compared using the Mann–Whitney U test. (B) Correlation between stage 3/2 ratio of sleep spindle density and age in children with ASD and CS. Correlation of data was tested using Pearson’s rank correlation coefficient. Stage 3/2 ≥1 group, children with ASD whose ratio of spindle density in stage 3 to stage 2 were ≥1; stage 3/2 <1 group, children with ASD whose ratio of spindle density in stage 3 to stage 2 were <1. Horizontal line inside the box, median; X inside the box, average; stage 3/2 ratio, the ratio of spindle density in stage 3 to stage 2; line, approximating curve of ASD; line with dot, approximating curve of CS; *, *p*-value < .05.

As shown in Figure 4, A, the stage 3/2 ≥1 group had a significantly younger age (5.0 ± 0.7 years, *p* < .05, ES *d* = 1.7, 1−β = 0.80) than the stage 3/2 < 1 group (7.1 ± 1.6 years). The stage 3/2 ratio of spindle density was correlated to age in the ASD group (*r* = −0.54, *p* = .04, 1−β = 0.60) but not in the CS group (*r* = 0.35, *p* = .21, 1−β = 0.25, Figure 4, B). Furthermore, the spindle density in stage 2 was significantly lower in the stage 3/2 ≥1 group (98.0 ± 73.8/h) than in the stage 3/2 <1 group (266.3 ± 121.1/h, *p* < .05, ES *d* = 1.68, 1−β = 0.99, Supplementary Figure 1A). The stage 3/2 ratio of spindle density was correlated to spindle density in stage 2 in the ASD group (*r* = −0.56, *p* = .04, 1−β = 0.64) but not in the CS group (*r* = 0.32, *p* = .27, 1−β = 0.21, Supplementary Figure 1B). There were no differences in the duration of stages 2, 3, and 4 in PSG between the stage 3/2 ≥1 and stage 3/2 <1 groups in stages 2–4 (Supplementary Table 2).

## Discussion

In this study, we quantified and compared spindle parameters across non-REM sleep stages between children with ASD and CS of children from Japan. Spindle parameters did not significantly differ between the groups but we found higher spindle density in stage 3 than in stage 2 cases in the ASD group.

The large-scale longitudinal study by Purcell et al. [7], which included 11 630 individuals, aimed to characterize normative distributions and epidemiological associations of spindle activity; they showed that spindle density increases with age during childhood and peaks around adolescence. Thus, the age of the participants in the present study may have affected the appearance of sleep spindles. Furthermore, previous reports have suggested that the number of sleep spindles is highly correlated with the IQ and that the relationship between these appears to be U-shaped, with positive and negative correlations for high (115–126) and low (77–107) IQ, respectively [13]. Therefore, the IQ level affects the results of the spindle analysis. To investigate the characteristics of sleep spindles in ASD in the present study, children with above-normal FSIQ/DQ and age range of childhood (4–10 years) were recruited to ensure a background as homogeneous as possible.

Between the ASD and CS groups, we observed no obvious differences in the sleep architecture, except for long REM sleep durations in the ASD group. However, Buckley et al. [39] reported decreased REM sleep in one-night PSG study and a lower nonverbal IQ ratio in children aged 2–13 years with ASD and a lower non-verbal IQ ratio (25.07–114.20) than in children with developmental delay (9.61–76.63) and TD children (80.57–142.81).

Previous studies of two-night PSG have demonstrated that sleep in the adaptation night was characterized by poorer sleep quality than in subsequent nights owing to increased sleep latency, less REM sleep, and frequent arousal [40–45]. Similar to adults, healthy children also showed improvements in sleep quality from the first to the second night [46]. We performed PSG for only one night and found differences in REM sleep but not in non-REM sleep. Considering children with ASD, a report of two-night PSG analysis in young children revealed that no expected second night increased the total sleep time or the REM sleep percentage, suggesting that a single-night PSG may be sufficient to evaluate children with ASD for TST or REM parameters [47]. However, the first-night effect could have influenced the non-REM sleep and the absence of group differences on the sleep spindle density in our study.

As shown in Figure 3, the average values of the spindle density, duration, and mean amplitude in C3 did not differ between the ASD and CS groups. Considering the frontal lobe, Farmer et al. [24] and Tessier et al. [23] reported reduced sleep spindle activities in children with ASD compared to those in TD children of an age range of 2–6 years with low-normal IQ and 6–13 years with normal IQ; however, Maski et al. [25] did not find differences in the children aged 9–16 years with normal IQ. Considering the central lobe, Farmer et al. [24] reported reduced sleep spindle activities; however, Tessier et al. [23] and Maski et al. [25] did not report any differences. Thus, compared to the frontal lobe, the spindle density in the central region reached the same level as TD earlier than the frontal robe. These differences in the spindle depend on the developmental discrepancy of the frontal and central regions; the development of the frontal lobe is nearly the last part of brain development [48] and the frontal and temporal lobe volumes changed at significantly slower rates in patients with autism than in the controls across the 2- to 11-year age range [49]. In our study, some PSG recordings were performed according to the standard methods described by the R&K criteria, including central and occipital EEG (C3, C4, O1, and O2) [50]. We did not include the F electrode in all the cases; thus, we analyzed only the C3 electrode. Based on the normal developmental state and age range [51], we speculated that the sleep spindle density in *F* is still lower in the ASD than in the control group; however, further investigation is warranted.

Furthermore, the distribution ranges were broader in the ASD than in the CS group, as shown in Figure 3, A. This may explain the heterogeneity and multifactorial neurodevelopmental characteristics of ASD [19]. For example, electrophysiological features may differ between the ASD subgroups with and without auditory hypersensitivity [52]. The appearance pattern of sleep spindles may also vary as ASDs are complex, pervasive, and heterogeneous and have multifactorial neurodevelopmental conditions [19]. This may also be the reason for the controversial findings of previous reports on ASD.

Nevertheless, the absence of a difference between the children with ASD and the CS of children could be a type II error. This should be examined by increasing the number of participants in the future.

Purcell et al. [7] reported changes in the spindle activity within the non-REM cycles involving >11 000 individuals; stage 2 spindle density was highest near the initiation of the first non-REM cycle and varies within the cycle dependent on age and spindle frequency but spindle density during N3 (stage 3 and 4) was unrelated to macroarchitecture. Moreover, it was reported that especially in younger individuals (aged <40 years; mostly children and adolescents), spindles occurred less often in N3 than in stage 2 sleep [7]. In our study, stage 2 spindle density in children with ASD was the highest in the first third of the sleep similar to Purcell’s study [7]; however, stage 3 spindle density was the highest in the last third of the sleep (Table 2). In addition, the stage 3/2 ratio of the spindle density was significantly higher in the ASD group in the last third of sleep (Figure 2, B).

In this study, we investigated the average values of spindle parameters and focused on the sleep stage-dependent appearance of sleep spindles. According to a previous study [6], the spindle densities and sigma activities in stage 2 are higher than those in slow-wave sleep in healthy participants. All of our CS of children showed the highest spindle density in stage 2, but five children with ASD showed a higher spindle density in stage 3 than in stage 2 (Figure 2, A). One case showed the highest spindle density even in stage 4 (# in Figure 2, A). After eliminating the effect of age, the significant difference in the stage 3/2 ratio between ASD and CS in whole sleep disappeared. However, significant differences remained in the last third (Figure 2, B). To the best of our knowledge, there have been no reports focusing on the sleep stage-dependent appearance of sleep spindles in children with ASD.

The findings provoked the following questions: “Despite the lower spindle density in stage 2, why did it not decrease in stage 3 in younger children with ASD?”; “Did delta waves intrude into stage 2, or alternatively, did sleep spindles continue to emerge among the delta waves in stage 3?” If delta waves appeared earlier in stage 2, the duration of stage 2 was plausibly shorter, but as shown in Supplementary Table 2, there were no differences in the duration of stages 2 and 3 between the two groups. Therefore, we concluded that in the stage 3/2 ≥1 group of children with ASD, the sleep spindles did not terminate and continued to emerge among delta waves in stage 3. To think especially about the case, which showed high spindle density in stage 4 (# in Figure 2, A), it may alternatively suggest a failure of spindle termination or that these additional sleep spindles originate from other brain regions.

The mechanism of spindle termination remains unclear [53]. The thalamic mechanisms of sleep spindle termination have been suggested to arise from suppression of rebound burst generation in TC cells after depolarization, generated by repetitively bursting TC cells [54] or from a gradual hyperpolarization of TRN cells during repeated cycles [55]. A computational study also proposed a cortical mechanism that could attenuate the driving role of corticothalamic inputs for thalamic spindle-like rhythms and contribute to their decline [56]. Additionally, insufficient secretion of other transmitters and hormones, such as serotonin and melatonin, could influence the pathophysiology of ASD [57, 58] and also sleep spindles; serotonin reuptake inhibitors increase the spindle density in N2 and N3 [59] and the oral administration of melatonin in humans enhances the power in the frequency range of the sleep spindle [60]. In ASD, secretion of these transmitters and hormones also differs individually; thus, various phenotypes could exist. These mechanisms may explain the relatively higher sleep spindle density in stage 3 in some children with ASD. In contrast, enhanced coupling of slow oscillations (SOs) to sleep spindles (SO-spindle coupling) may be connected to the generation of sleep spindles from different origins. A topographical study revealed that SO-spindle coupling in central (Cz) and frontal (Fpz) electrodes is evident during N3 sleep [61]. Even though sleep spindle deficits in patients with schizophrenia have been reported [16], SO-spindle coupling is not affected in this population [62]. A similar phenomenon may also occur in some children with ASD.

In the current study, we found the possibility of the characteristics of the stage 3/2 ≥1 group of children with ASD who were younger and demonstrated lower spindle densities in stage 2 than those in the ASD stage 3/2 <1 and CS groups. The stage 3/2 ratio of spindle density was negatively correlated with age in children with ASD but had no obvious correlation in CS of children (Figure 4, B). As spindles are generated in the TRN, GABAergic neurons are primarily involved [63]. In ASD, reductions in GABA levels [64] and numbers of GABAergic neurons [65] have been reported. Interestingly, Horder et al. suggested that GABA abnormalities in ASD cases are age-dependent [66]. From these lines of evidence, lower spindle densities in stage 2 in younger children with ASD and relatively lower IQs may originate from immature and/or pathological GABAergic systems. In addition, GABAergic neurons in the highly interconnected TRN act as pacemakers and are both necessary and sufficient for spindle generation [67]. The entire corticothalamic network allows widespread synchronization of non-REM sleep, and the simultaneous appearance of spindles requires intact TC and corticothalamic connections [68].

As sleep spindle deficits have been confirmed in several groups of patients with chronic, medicated schizophrenia [16], several studies have reported that the presence of ASD or autistic traits might be a significant predictor of psychotic episodes [69]. Approximately half of the cases with a clinical and research diagnosis of schizophrenic psychosis had ASD according to results obtained from parental interviews using the Diagnostic Interview for Social and Communications Disorders [70]. Several reports have indicated dopaminergic and glutaminergic dysfunctions in both ASD and schizophrenia [71–75]. Lower spindle densities may reflect similar neuropathological statuses in ASD and schizophrenia.

Early studies correctly predicted brain wave organization and maturation differences in ASD [76, 77]. A diffusion tensor imaging study on healthy adults reported that individuals with higher spindle power had higher axial diffusivity in the temporal lobe and in the tracts in and surrounding the thalamus [78]. These results indicated that sleep spindles reflect the localized microstructural properties of white matter tracts. Theoretical and empirical work is beginning to reveal that ASD is associated with a complex functional phenotype, characterized by insufficient connectivity of large-scale brain systems [79]. Longitudinal data have shown slower white matter growth among boys with autism [80]; in particular, aberrant thalamofrontal connections were reported in high-functioning ASD cases [81]. Spindle duration and density change with brain maturation [32]. There are no reports concerning age-related changes in TC connectivity in children with ASD; however, our stage 3/2 ≥1 group was younger (Figure 4, A), and there are no age-dependent changes reported in spindle density in TD children [7, 32].

Hyperpolarization of TC cells along with the deepening of non-REM sleep stages modifies the EEG from low-voltage fast activity to theta with spindles and, then, with further hyperpolarization, to delta activity [63]. The amplitude and slope of the slow wave were proposed as markers of synaptic density and strength in the TC loop [82]; thus, N3 sleep is thought to be a proxy of cortical maturation [83]. Recent studies have focused on the relationship between sleep spindles and N3 sleep in children with ASD to assess the possibility that hyperpolarization of the thalamocortical loop is not fully functioning in the ASD group [84, 85]. Lambert et al. [84] reported a decrease in sleep spindle in N2 and a decrease in N3 sleep in the first third of sleep in children with ASD. Mylonas et al. [85] reported disruption of SO-spindle coupling during N2 in children and adolescents with ASD. In our ASD group, the N3 spindle density markedly increased in the last third of sleep, especially in young children. Previous reports [84, 85] and our data suggest that the capacity of the ASD group to hyperpolarize the TC loop is immature and there may be a possibility of not maintaining hyperpolarization until the last third of sleep.

Growth of the frontal lobe is finally accelerated at adolescence in TD children [51]; it grows even more slowly in children with ASD [49, 86]. Shinomiya et al. [87] and Scholle et al. [32] suggested that frontal spindle activity could be a good indicator of biological maturation for evaluating the development process in the central nervous systems of children and adolescents. Considering the result of a former report that found low spindle density in the frontal electrodes in children with ASD [23, 24], and our study, which showed higher spindle density in stage 3 than in stage 2 in the last third of sleep in children with ASD, we should focus on the frontal lobe sleep spindles and also pay attention to the sleep stage-dependent appearance of sleep spindles in children with ASD, especially in the later part of the sleep period.

## Limitations

In this preliminary study, the sample size was very small because of the fundamental difficulty of performing PSG in children with ASD. Although non-significant, the ASD group showed male preponderance and consisted of younger individuals. We could not conduct WISC to evaluate the children owing to time constraints. As PSG was performed only on one night because of ethical issues, a first-night effect cannot be ruled out. However, it has been reported that the spectral properties of sleep EEG, including power in the frequency range of the sleep spindle (7–16 Hz), are highly conserved within individuals across nights [88].

Finally, based on the AASM scoring rules for children, the two temporospatially distinct types of sleep spindles may have existed [3]. However, we only analyzed data from C3 as fast and slow spindles because we could not apply more electrodes, such as F1/2 or F3/4, where slow spindles appear most distinctively in children with ASD who are often hypersensitive. As a previous report identified a higher power of frontal slow sleep spindles in children with both ADHD and ASD [17], we should consider frontal slow sleep spindles in future studies.

Nevertheless, our findings suggested that spindle density, duration, and mean amplitude do not significantly differ between children in the ASD and CS groups. When the ASD group was divided into the stage 3/2 ≥1 group and the stage 3/2 <1 group, the former group represented an abnormal generation and termination of spindles due to insufficient maturation of the TRN and the TC network. Further large-scale prospective studies are needed to validate our findings.

## Supplementary Material

zpac037_suppl_Supplementary_Material

zpac037_suppl_Supplementary_Figure_S1

## Data Availability

The data underlying this article are not available to be shared due to ethical and institutional restrictions.
