# Supplementary material for: A preliminary study of sleep spindles across non-rapid eye movement sleep stages in children with autism spectrum disorder
Source: Sleep Adv. 2022 Oct 20;3(1):zpac037. doi: 10.1093/sleepadvances/zpac037 (PMC10104411; doi:10.1093/sleepadvances/zpac037)
Supplement: zpac037_suppl_Supplementary_Material [file zpac037_suppl_supplementary_material.docx]

**A preliminary study of sleep spindles** **across non-REM sleep stages in children with** **autism spectrum disorder**

Midori Kawahara, MD,^1,2^ Kuriko Kagitani-Shimono, MD, PhD,^1^ Kumi Kato-Nishimura, MD, PhD,^1,2^ Noboru Ohki,^3^ Masaya Tachibana, MD, PhD,^1,2^ Takafumi Kato, DDS, PhD,^4^ Masako Taniike, MD, PhD,^1,2^ Ikuko Mohri, MD, PhD^1,2^

1. Department of Child Development, United Graduate School of Child Development, Osaka University, Suita, Osaka, Japan

2. Department of Pediatrics, Osaka University Graduate School of Medicine, Suita, Osaka, Japan

3. NoruPro Light Systems Incorporation, Kokubunji-shi, Tokyo, Japan

4. Department of Neuroscience and Oral Physiology, Graduate School of Dentistry, Osaka University, Suita, Osaka, Japan

Corresponding author: Ikuko Mohri, MD, PhD, Department of Child Development, United Graduate School of Child Development, Osaka University, 2-2, Yamadaoka, Suita, Osaka, 5650871, Japan.

E-mail address: ikuko@kokoro.med.osaka-u.ac.jp (I. Mohri).

Supplementary Figure 1. Characteristics of spindle density in stage 2

A. Comparison based on stage 2 spindle density between the stage 3/2 ratio ≥1 and stage 3/2 ratio <1 groups. The data were compared using the Mann–Whitney U test.

B. Correlation between stage 3/2 ratio of sleep spindle density and stage 2 spindle density in children with ASD and CS. Correlation of data was tested using Pearson’s rank correlation coefficient. stage 3/2 ≥1 group, children with ASD whose ratio of spindle density in stage 3 to stage 2 were ≥1; stage 3/2 <1 group, children with ASD whose ratio of spindle density in stage 3 to stage 2 were <1; horizontal line inside the box, median; X inside the box, average; stage 3/2 ratio of sleep spindle density, the ratio of spindle density in stage 3 to stage 2; line, approximating curve of ASD; line with dot, approximating curve of CS; *, p-values <0.05

Supplementary Figure 1

Supplementary Table 1. Differences in stages 2, 3, and 4 durations among the ASD and CS groups

| Duration  (%) | | First third of the sleep | | | Second third of the sleep | | | Last third of the sleep | | |
| --- | --- | --- | --- | --- | --- | --- | --- | --- | --- | --- |
|  |  | Mean±SD | p | ES d1-β | Mean±SD | p | ES d  1-β | Mean±SD | p | ES d  1-β |
| Stage 1 | ASD | 12.25±6.36 | 0.19 | 0.51  0.25 | 11.52±5.85 | 0.83 | 0.08  0.06 | 16.55±7.86 | 0.11 | 0.63  0.37 |
|  | CS | 8.89±6.82 |  |  | 10.89±8.78 |  |  | 11.38±8.44 |  |  |
| Stage 2 | ASD | 30.18±11.05 | 0.25 | 1.00  0.72 | 42.30±11.77 | 0.15 | 0.56  0.29 | 41.33±10.54 | 0.19 | 0.50  0.25 |
|  | CS | 25.95±7.59 |  |  | 49.57±14.23 |  |  | 47.78±14.72 |  |  |
| Stage 3 | ASD | 11.03±10.57 | 0.36 | 0.35  0.15 | 5.10±3.43 | 0.95 | 0.03  0.05 | 6.04±5.14 | 0.42 | 0.31  0.12 |
|  | CS | 8.29±2.80 |  |  | 5.00±4.17 |  |  | 4.71±3.27 |  |  |
| Stage 4 | ASD | 38.84±15.00 | 0.02 | 0.99  0.71 | 13.71±7.07 | 0.21 | 0.49  0.24 | 2.25±3.73 | 0.22 | 0.47  0.23 |
|  | CS | 52.01±11.39 |  |  | 10.31±6.80 |  |  | 4.39±5.20 |  |  |
| REM | ASD | 7.72±6.81 | 0.15 | 0.56  0.30 | 27.39±9.51 | 0.25 | 0.44  0.20 | 33.83±6.92 | 0.29 | 0.41  0.18 |
|  | CS | 4.63±3.85 |  |  | 23.50±7.93 |  |  | 30.89±7.36 |  |  |

Data are presented as means±SD. SD, standard deviation; REM, rapid eye movement ASD, autism spectrum disorder; CS, community samples; p, p-value; ES d, effect size d; 1-β, power.

Supplementary Table 2. Differences in stages 2, 3, and 4 durations among the ASD stage 3/2 ≥1 group, ASD stage 3/2 ˂1 group, and CS group

|  | ASD | ASD | CS | Stage 3/2 ≥1 group vs. stage 3/2*˂*1 group | | | Stage 3/2 ≥1 group vs. CS group | | |
| --- | --- | --- | --- | --- | --- | --- | --- | --- | --- |
|  | Stage 3/2 ≥1 group | Stage 3/2 *˂*1 group |  |  |  |  |  |  |  |
|  |  |  |  | p | ES d | 1-β | p | ES d | 1-β |
| Number of participants | n=5 | n=9 | n=14 |  |  |  |  |  |  |
| Age (years)^a^ | 5.0±0.7 | 7.1±1.6 | 7.6±1.5 | 0.02 | 1.7 | 0.80 | 0.002 | 2.22 | 0.98 |
| Stage 2 duration (%)^a^ | 32.9±9.3 | 40.9±7.0 | 40.9±9.0 | 0.09 | 0.97 | 0.36 | 0.11 | 0.87 | 0.35 |
| Stage 3 duration (%)^a^ | 6.0±1.1 | 8.5±5.8 | 6.4±2.3 | 0.37 | 0.60 | 0.17 | 0.63 | 0.22 | 0.07 |
| Stage 4 duration (%)^a^ | 20.5±2.3 | 16.5±7.6 | 22.3±4.6 | 0.29 | 0.71 | 0.22 | 0.41 | 0.15 | 0.49 |

Data are presented as means±SD. SD, standard deviation; ASD, autism spectrum disorder; CS, community samples; stage 3/2 ≥1 group, children with ASD whose ratio of spindle density in stage 3 to stage 2 was ≥1; stage 3/2 ˂1 group, children with ASD whose ratio of spindle density in stage 3 to stage 2 was ˂1; p, p-value; ES d, effect size d; 1-β, power; a: Student’s t-test.
