# Supplementary figures and images for: A preliminary study of sleep spindles across non-rapid eye movement sleep stages in children with autism spectrum disorder
Source: Sleep Adv. 2022 Oct 20;3(1):zpac037. doi: 10.1093/sleepadvances/zpac037 (PMC10104411; doi:10.1093/sleepadvances/zpac037)

**A**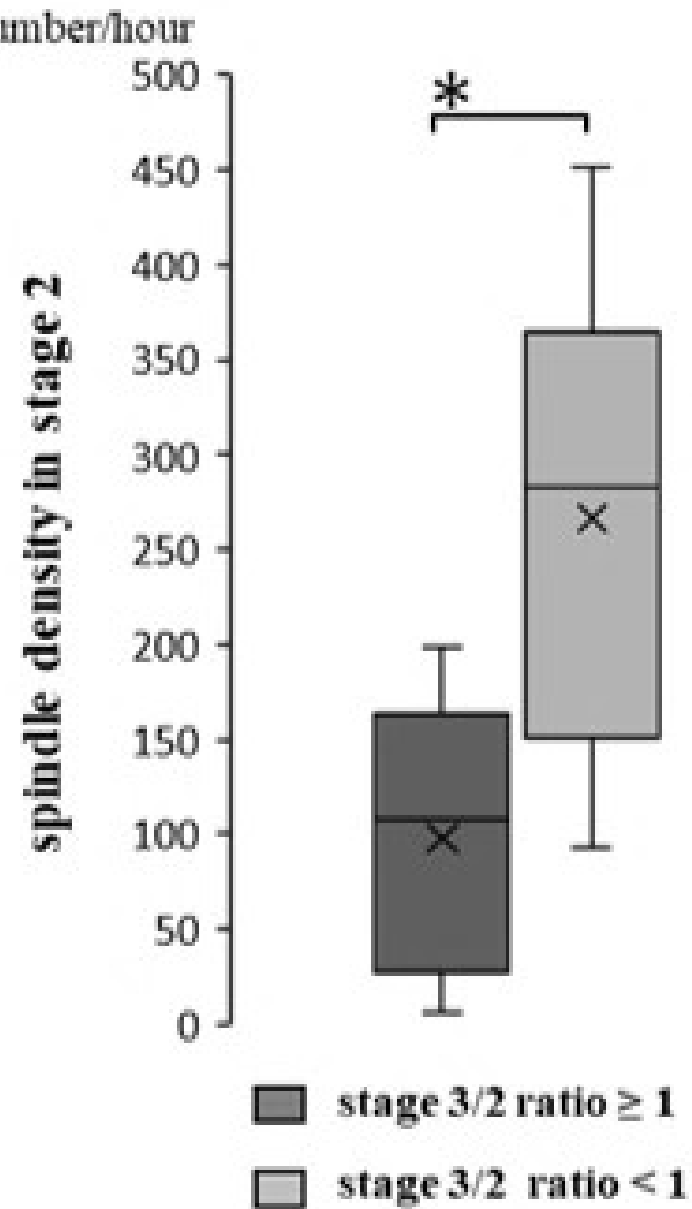**B**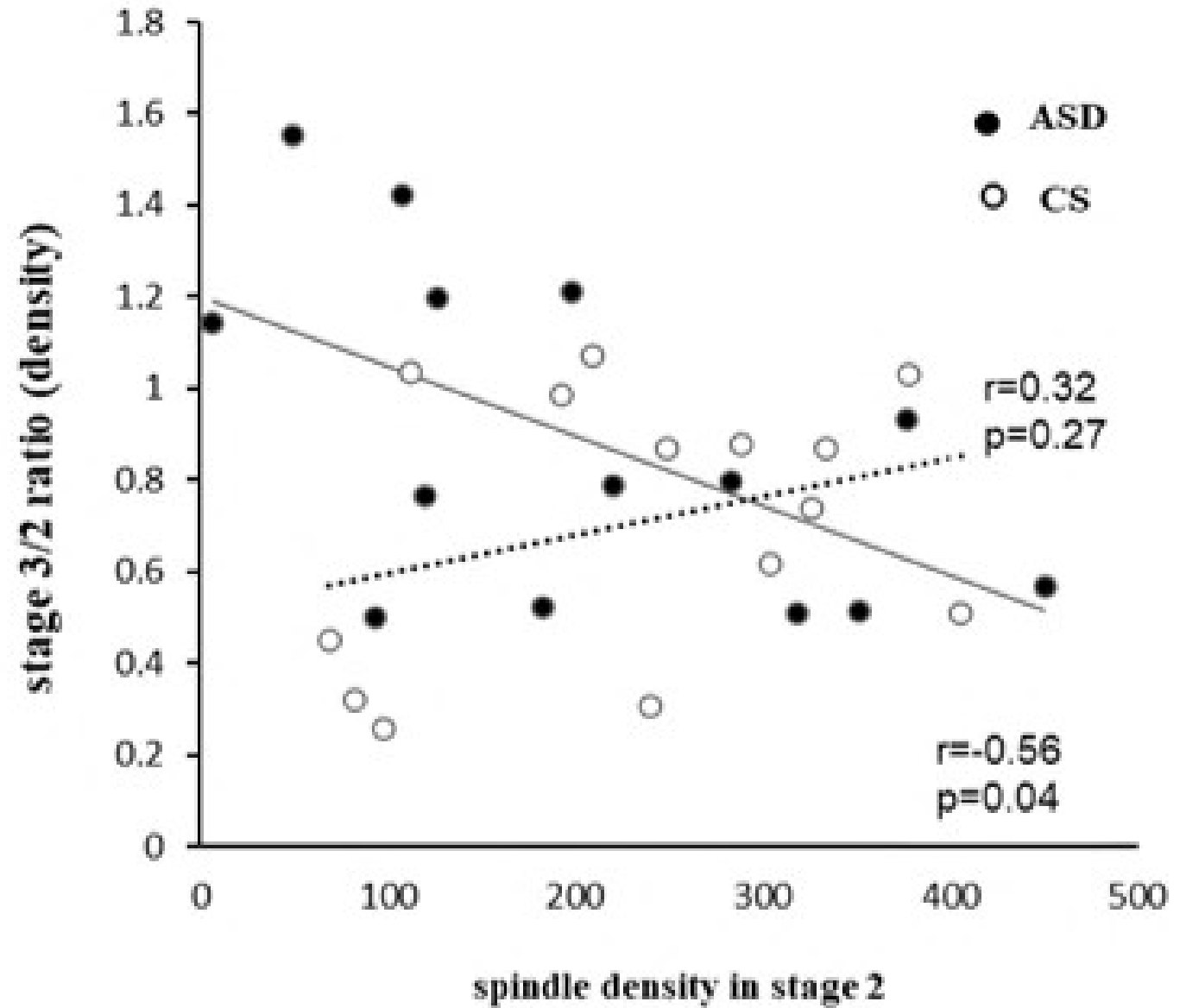

Supplement: zpac037_suppl_Supplementary_Figure_S1 [file zpac037_suppl_supplementary_figure_s1.pdf]
